# Macrolide-Resistant *Mycoplasma pneumoniae* Infections among Children after COVID-19 Pandemic, Ohio, USA

**DOI:** 10.3201/eid3103.241570

**Published:** 2025-03

**Authors:** Amy L. Leber, Tori Embry, Kathy Everhart, Jeanette Taveras, Sophonie J. Oyeniran, Huanyu Wang

**Affiliations:** Author affiliations: Nationwide Children’s Hospital, Columbus, Ohio, USA (A.L. Leber, T. Embry, K. Everhart, J. Taveras, S.J. Oyeniran, H. Wang); The Ohio State University, Columbus (A.L. Leber, J. Taveras, S.J. Oyeniran, H. Wang)

**Keywords:** Mycoplasma pneumoniae, COVID-19, respiratory infections, severe acute respiratory syndrome coronavirus 2, SARS-CoV-2, SARS, coronavirus disease, viruses, coronavirus, macrolide resistance, antimicrobial resistance, pediatrics, Ohio, United States

## Abstract

*Mycoplasma pneumoniae *infections decreased in Ohio, USA, during the COVID-19 pandemic but reemerged in 2023; >2,000 cases were reported during September 2023­–September 2024. Of 995 *M. pneumoniae*–positive samples, 24 (2.4%) had mutations for macrolide-resistant *M. pneumoniae* (MRMp). MRMp rates are low but increasing. MRMp surveillance is crucial for monitoring antimicrobial resistance.

*Mycoplasma pneumoniae* is a major pathogen of community-acquired respiratory infection in school-age children, accounting for 10%–40% of community-acquired pneumonia among hospitalized children (*1*). *M. pneumoniae* is endemic worldwide, and epidemics occur every few years (*1*). During the COVID-19 pandemic, public health measures taken to reduce transmission of SARS-CoV-2 also decreased *M. pneumoniae*, and we saw almost no *M. pneumoniae* activity in pediatric patients at Nationwide Children’s Hospital (NCH), Columbus, Ohio, USA. Since the fall of 2023, reports of *M. pneumoniae* infection have increased worldwide (*2*). In central Ohio, we observed a reemergence of *M. pneumoniae* activity in children beginning in September 2023 and a sharp increase in the summer of 2024.

Macrolides are the drug of choice for treating *M. pneumoniae* infections (*3*). Macrolide resistance is conferred by point mutations within the V region of 23S rRNA, which interferes with bacterial protein synthesis leading to organism death. The most common mutation is the change of A to G at location 2063 of the gene (A2063G), accounting for >95% of the *M. pneumoniae* variants in the United States, along with the A2064G mutation (*4*). During 2015–2018, macrolide-resistant *M. pneumoniae* (MRMp) rates in the United States ranged from 2.1% to 18.3% (*5*); in a similar period, we reported a 2.8% MPMp rate in our pediatric population (*6*). With the reemergence of *M. pneumoniae*, we sought to determine the rate of MRMp infections in children in central Ohio.

## The Study

The microbiology laboratory at NCH offers 2 tests to detect *M. pneumoniae*: FilmArray Respiratory Panel version 2.1 (RP2.1; BioFire Diagnostics, https://www.biofiredx.com) (*7*) and a standalone laboratory-developed PCR (*8*,*9*). During September 1, 2023­–September 30, 2024, we identified patients <21 years of age testing positive for *M. pneumoniae* by RP2.1 or PCR. Patient sample collection occurred in inpatient, outpatient, and emergency department (ED) settings (Appendix Table 1). We retrieved a subset of remnant specimens for further characterization, as previously described (*6*) (Appendix). 

We collected data on patient demographics, symptoms, clinical and laboratory findings, and hospitalization status from electronic health records. We analyzed age by Kruskal-Wallis test and reported medians and interquartile ranges (IQRs). We analyzed categorical variables by χ^2^ test and conducted analyses by using GraphPad (GraphPad Software Inc., https://www.graphpad.com). 

During the study period, the NCH microbiology laboratory performed 18,035 tests and identified 2,616 (14.5%) *M. pneumoniae*–positive samples from 2,469 unique patients during 2,478 medical encounters (Table 1; Figure). *M. pneumoniae* positivity rates remained steady during September 2023–May 2024, then rose sharply in early June 2024. The median age of *M. pneumoniae*–positive patients was 8.8 (IQR 5.8–11.6) years; 1,317 (53.3%) were male and 1,152 (46.7%) female (Table 2). Among patients, 304 (12.3%) were hospitalized and 53 (2.1%) required intensive care unit (ICU) admission. Among the 359 *M. pneumoniae*–positive patients who had RP2.1 testing or RP2.1 and PCR testing, 129 (35.9%) had codetection of other respiratory pathogens on the panel; all were viruses (Table 2). The most common codetections were rhinovirus/enterovirus (n = 93, 72.0%) and adenovirus (n = 15, 11.6%).

**Table 1:** Dates and characteristics of testing for macrolide-resistant *Mycoplasma pneumoniae* infections among children after COVID-19 pandemic, Ohio, USA*

Date	Total no. tested	*M. pneumoniae*­–positive, no. (%)	No. (%) sequenced	Macrolide-resistant, no. (%)
2023				
Sep	818	5 (0.6)	0	NA
Oct	1,001	29 (2.9)	0	NA
Nov	1,348	59 (4.4)	0	NA
Dec	1,697	62 (3.7)	0	NA
2024				
Jan	1,388	57 (4.1)	6 (10.5)	0
Feb	1,303	29 (2.3)	6 (20.7)	0
Mar	1,085	45 (4.2)	1 (2.2)	0
Apr	965	56 (5.8)	27 (48.2)	0
May	1,045	106 (10.1)	47 (44.3)	0
Jun	1,020	266 (28.1)	145 (54.1)	1 (0.7)
Jul	1,318	407 (30.9)	226 (55.5)	2 (0.9)
Aug	2,009	642 (32.0)	285 (44.4)	10 (3.9)
Sep	3,038	853 (28.1)	252 (29.5)	11 (4.4)
Total	18,035	2,616 (14.5)	995 (38.0)	24 (2.4)

*NA, not applicable.

**Figure:**
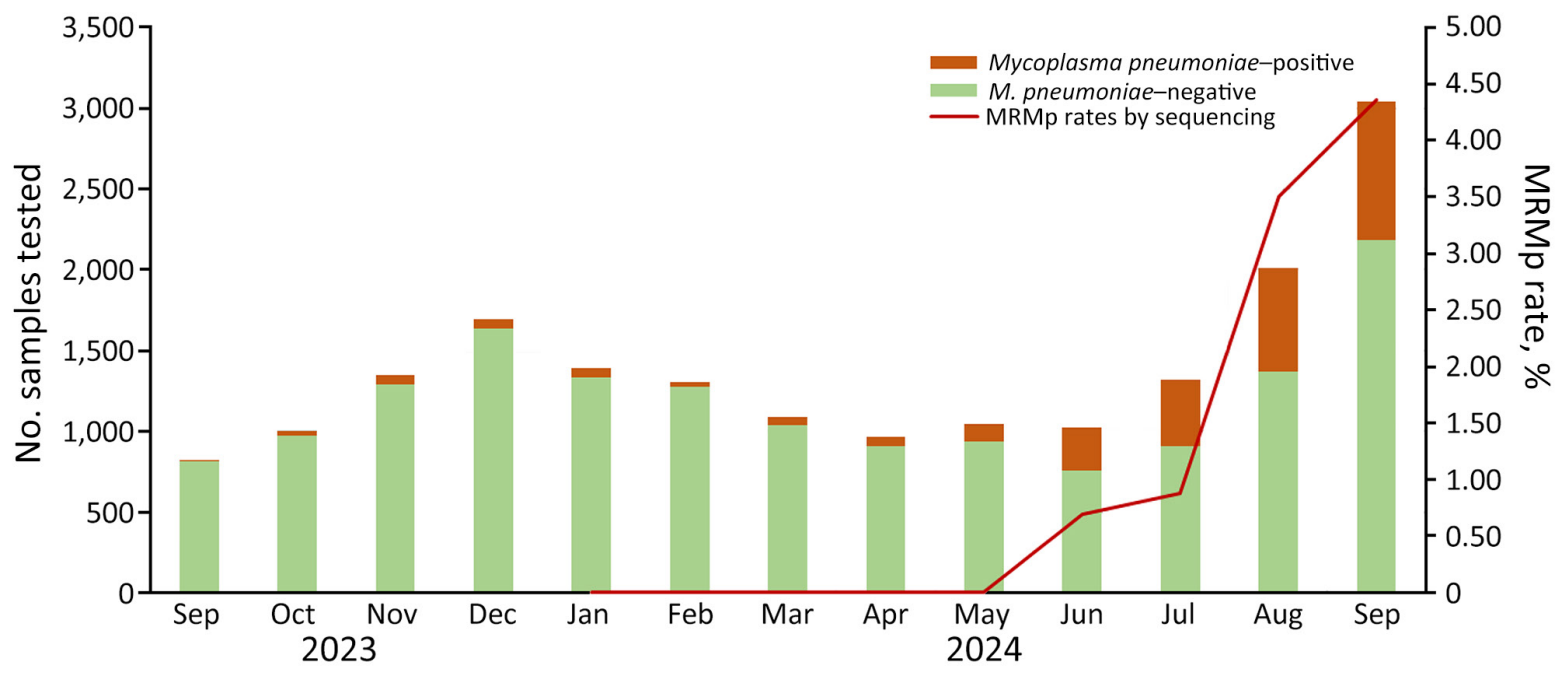
Monthly testing volumes and rates of MRMp infections among children after COVID-19 pandemic, Ohio, USA. Samples were tested for *Mycoplasma pneumoniae* infection during September 2023–September 2024 by FilmArray Respiratory Panel version 2.1 (BioFire Diagnostics, https://www.biofiredx.com), an in-house *M. pneumonia* PCR, or both. Macrolide resistance was determined in a subset of samples. MRMp rates were not available during September 2023–December 2023. MRMp, macrolide-resistant *Mycoplasma pneumoniae*.

**Table 2:** Laboratory characteristics for macrolide-resistant *Mycoplasma pneumoniae* infections among children after COVID-19 pandemic, by age group, Ohio, USA*

Characteristics	Total no.	Age group, y				p value
		<2	2 to <6	6 to <10	>10	
No. samples tested	18,035	5,288	4,373	3,401	4,973	NA
* M. pneumoniae*­–positive	2,616 (14.5)	109 (2.1)	601 (13.7)	904 (26.6)	1,002 (20.1)	<0.0001
Sequenced	995 (38.0)	30 (27.5)	224 (37.3)	363 (40.2)	378 (37.7)	NA
Macrolide-resistant	24 (2.4)	1 (3.3)	6 (2.7)	12 (3.3)	6 (1.6)	NS
No. positive unique patients	2,469	82	572	865	950	NA
Sex						
M	1,317 (53.3)	48 (58.5)	319 (55.8)	457 (52.8)	493 (51.9)	NS
F	1,152 (46.7)	34 (41.4)	253 (44.2)	408 (47.2)	457 (48.1)	
No. medical encounters	2,478	83	575	866	954	NA
Hospitalization	304 (12.3)	23 (27.7)	91 (15.8)	81 (9.4)	109 (11.4)	<0.0001
Intensive care unit admission	53 (2.1)	6 (7.2)	19 (3.3)	14 (1.6)	14 (1.5)	0.0009
RP2.1 tested	359	31	98	100	130	NA
Codetection†	129 (35.9)	19 (61.3)	51 (52.0)	31 (31.0)	28 (21.5)	<0.0001

*Values are no. (%) except as indicated. NA, not applicable; NS, not statistically significant; RP2.1, FilmArray Respiratory Panel version 2.1 (BioFire Diagnostics, https://www.biofiredx.com). †Codetection of *M. pneumoniae* and other viruses.

During January 2024–September 2024, we attempted to sequence 1,096 (41.9%) of 2,616 positive samples and successfully sequenced 995 (91%). Among successfully sequenced samples, 85 (35.4%) were from inpatients, 787 (39.8%) from outpatients, and 123 (30.9%) from ED patients. We detected mutations associated with MRMp in 24 (2.4%) samples; 22 were A2063G, 1 A2064G, and 1 A2064T. The percentage of resistance detected differed by month, and the highest rate (4.4%) was detected in September 2024 (p = 0.0466) (Table 1; Figure). The median age of those 24 patients was 8.4 (IQR 5.0–9.8) years; 15 (62.5%) were male and 9 (37.5%) female. Among MRMp–positive patients, 5 (20.8%) had previous azithromycin exposure, and 5 required hospitalization (Appendix Table 2). Two patients had another *M. pneumoniae*–positive sample collected 3–4 weeks before the sample from which mutations were detected. In both cases, we sequenced the prior sample and detected no mutations. Those 2 patients received azithromycin at both of their clinical encounters.

*M. pneumoniae* positivity rates were significantly higher among children >6 years of age (p<0.0001). The rate among children <2 years of age was 2.1% compared with 26.6% among children 6–10 years of age. In contrast, the hospital and ICU admission rates were higher for children <2 years of age (p<0.001) (Table 2). Younger children also had higher rates of codetection; 61.3% of children <2 years of age had other respiratory pathogens detected (p<0.001) (Table 2). Mutations were detected in all age groups.

During the COVID-19 pandemic, introduction of nonpharmaceutical interventions interrupted epidemics of other respiratory pathogens, resulting in a substantial decline of respiratory infections worldwide (*10*). Since 2021, other respiratory pathogens have resurged after those interventions were lifted and community transmission returned (*11*). However, we detected little *M. pneumoniae* activity in our patient population until September 2023. That delayed reemergence has also been reported from other parts of the world (*11*). Unlike other areas where *M. pneumoniae* has reemerged with case numbers similar to or slightly higher than prepandemic times (*12*,*13*), the ongoing *M. pneumoniae* surge in our patient population is the largest we have seen in the past 10 years, >2,000 cases in 4 months (June 2024–September 2024), compared with 1,350 total cases during January 2012–January 2019. 

Although more children were infected with *M. pneumoniae* in 2024, the hospitalization and ICU admission rates were lower than our previous prepandemic report (*6*). That reduction is possibly because of the increased availability of molecular testing and greater awareness of *M. pneumoniae* testing during periods of heightened activity; 70% of testing orders came from outpatient or ED visits. The progression and severity of this *M. pneumoniae* reemergence has yet to be evaluated.

Surveillance data from the Centers for Disease Control and Prevention (https://www.cdc.gov/mycoplasma/php/surveillance) suggest that the 2024 *M. pneumoniae* surge involved more young children (2–4 years). However, incidence of *M. pneumoniae* infection in our cohort remained highest among school-age children and adolescents. The median age of *M. pneumoniae*–positive children in this study was similar to our previous prepandemic report (*6*). Other countries also observed higher detection among school-age children and adolescents during the 2023–2024 *M. pneumoniae* surge (*12*,*14*).

MRMp has been reported globally and rates vary between regions. Few data are available in the United States, particularly after the COVID-19 pandemic. One report from southeast Germany showed a 2.6% resistance rate among 2023–2024 *M. pneumoniae* strains (*15*); another study from southern China found a 96.4% resistance rate after COVID-19 (*12*). We found that the MRMp rate remains low in this study population; only 2.4% of detected *M. pneumoniae* carried the mutation. However, MRMp rates increased in September 2024 (4.4%) compared with June 2024 (0.7%) and May 2024 (n = 0) (p = 0.0466). We sequenced 38.0% of *M. pneumoniae*–positive samples across all age groups and clinical settings, thus reflecting MRMp rates across the patient population. More work is needed to understand MRMp in different patient populations and geographic locations and its effects on patient care.

One limitation of this study is that it was a single-center study; thus, MRMp rates might not reflect other US regions in or different populations. The data may continue to evolve because the *M. pneumoniae* surge is ongoing.

## Conclusions

In summary, we report macrolide resistance in *M. pneumoniae* after COVID-19 in our community. Although MRMp remains low, MRMp is trending upward, underscoring the need for vigilant surveillance to provide accurate information for management of children with *M. pneumoniae* infection and maintain awareness of antimicrobial resistance.

Appendix Additional information on macrolide-resistant *Mycoplasma pneumoniae* among children after COVID-19 pandemic, Ohio, USA.
