# Supplementary material for: Macrolide-Resistant Mycoplasma pneumoniae Infections among Children after COVID-19 Pandemic, Ohio, USA
Source: Emerg Infect Dis. 2025 Mar;31(3):555–8. doi: 10.3201/eid3103.241570 (PMC11878328; doi:10.3201/eid3103.241570)
Supplement: Appendix — Additional information on macrolide-resistant Mycoplasma pneumoniae among children after COVID-19 pandemic, Ohio, USA. [file 24-1570-Techapp-s1.pdf]

Article DOI: https://doi.org/10.3201/eid3103.241570

EID cannot ensure accessibility for supplementary materials supplied by authors. Readers who have difficulty accessing supplementary content should contact the authors for assistance.

Macrolide-Resistant *Mycoplasma pneumoniae* Infections among Children after COVID-19 Pandemic, Ohio, USA

Appendix

Additional Methods

In brief, we extracted nucleic acids from samples, then amplified and sequenced for a 191-bp region of *Mycoplasma pneumoniae* domain V of the 23S rRNA (nt 1937–2154; GenBank accession no. X68422). We compared the sequences were with the corresponding region of the wildtype reference strain (ATCC accession no. 15322) to detect mutations at locations 2063 and 2064 of the 23 rRNA gene.

Appendix Table 1. *Mycoplasma pneumoniae* testing volume, number of positives, sequencing volume and resistance detection by month and location.

	No. tested				*M. pneumoniae*–positive inpatients			*M. pneumoniae*–positive outpatients			*M. pneumoniae*–positive ED patients		
Date	Total	IP	OP	ED	Total	No. sequenced (%)	No. resistant (%)	Total	No. sequenced (%)	No. resistant (%)	Total	No. sequenced (%)	No. resistant (%)
2023													
Sep	818	284	298	236	2	0	NA	1	0	NA	2	0	NA
Oct	1,001	379	361	261	8	0	NA	16	0	NA	5	0	NA
Nov	1,348	409	576	363	14	0	NA	34	0	NA	11	0	NA
Dec	1,697	487	725	485	13	0	NA	35	0	NA	14	0	NA
2024													
Jan	1,388	449	543	396	15	1 (6.7)	0	30	5 (16.7)	0	12	0	0
Feb	1,303	431	501	371	9	1 (11.1)	0	14	5 (35.7)	0	6	0	0
Mar	1,085	352	352	381	9	1 (11.1)	0	22	0	0	14	0	0
Apr	965	342	292	331	19	8 (42.1)	0	29	16 (55.2)	0	8	3 (37.5)	0
May	1,045	384	334	327	26	13 (40.0)	0	64	29 (45.3)	0	16	5 (31.3)	0
Jun	1,020	258	466	296	35	15 (42.9)	0	179	106 (59.2)	1 (0.9)	52	24 (46.2)	0
Jul	1,318	287	744	287	29	18 (62.1)	0	329	187 (56.8)	2 (1.1)	49	21 (42.9)	0

Date	No. tested				*M. pneumoniae*–positive inpatients			*M. pneumoniae*–positive outpatients			*M. pneumoniae*–positive ED patients		
	Total	IP	OP	ED	Total	No. sequenced (%)	No. resistant (%)	Total	No. sequenced (%)	No. resistant (%)	Total	No. sequenced (%)	No. resistant (%)
Aug	2,009	340	1,289	380	30	16 (53.3)	0	525	239 (45.5)	8 (3.4)	87	30 (34.5)	2 (6.7)
Sep	3,038	372	2,065	601	31	12 (38.7)	2 (16.7)	700	200 (28.6)	7 (3.5)	122	40 (32.8)	2 (5.0)
Total	18,035	4,774	8,546	4,715	240	85 (35.4)	2 (2.4)	1,978	787 (39.8)	18 (2.3)	398	123 (30.9)	4 (3.3)

*ED, emergency department; IP, inpatient; NA, not applicable; OP, outpatient.

Appendix Table 2. Summary of clinical findings for patients testing positive for macrolide-resistant *Mycoplasma pneumoniae*

Patient no.	Age,y/sex	Underlying conditions	Signs and symptoms	Clinical diagnosis	Hospitalization, initial testing	Prior exposure to azithromycin	Definitive treatment	Follow up
1	1.8/M	None	Congestion, cough	URI	No	No	Azithromycin	NA
2	5.6/M	None	Cough, shortness of breath, emesis	URI	No	No	Azithromycin	NA
3	10/M	None	Fever, cough, emesis, diarrhea	URI	No	No	Azithromycin	Improved
4	9.9/M	None	Fever, cough, rash	Pneumonia due to *M. pneumoniae*	No	Yes	Azithromycin & Amoxicillin/clavulanate	Improved
5	9.9/F	None	Recurrent cough	URI	No	Yes	Azithromycin	Resolved
6	3.6/M	None	Fever, cough	Pneumonia due to *M. pneumoniae*	No	No	Azithromycin	Ongoing cough with focal lung findings
7	9.3/M	None	Fever, cough, headache	Lower respiratory tract infection	No	No	Azithromycin	Admitted at day 3 of therapy due to hypoxia
8	12.3/M	None	Fever, cough	Multifocal pneumonia	No	Yes	Cefdinir	NA
9	6.7/F	Obesity, autism spectrum disorder	Cough	Pneumonia	No	No	Azithromycin & Amoxicillin	Improved
10	3.9/F	None	Fever, cough, sore throat	Pneumonia due to *M. pneumoniae*	No	No	Azithromycin	NA
11	8.9/F	Asthma	Cough	Mild asthma exacerbation	No	No	Azithromycin	NA
12	7.1/M	None	Fever, cough, congestion, sore throat, headache	Left lower lobe pneumonia	No	No	Azithromycin & Amoxicillin	Ongoing cough. Developed acute otitis media
13	7.1/F	None	Fever, cough, increased work of breathing	*M. pneumoniae* pneumonia, respiratory failure	Yes, ICU-level care	No	Azithromycin	Discharged
14	3.5/M	None	Fever, congestion,	*M. pneumoniae* pneumonia, respiratory failure, tonsillitis	Yes, ICU-level care	No	Azithromycin followed with levofloxacin	Discharged

Patient no.	Age,y/sex	Underlying conditions	Signs and symptoms	Clinical diagnosis	Hospitalization, initial testing	Prior exposure to azithromycin	Definitive treatment	Follow up
15	4.4/F	None	Fever, headache, stomachache	Strep throat, *M. pneumoniae*	No	No	Azithromycin	Resolved
16	4.8/M	None	Cough, congestion, aches	*M. pneumoniae* infection	No	Yes	Azithromycin	NA
17	6.8/M	None	Fever cough	*M. pneumoniae* infection	No	No	Azithromycin	NA
18	7.5/M	Febrile seizure	Fever, cough, congestion, sore throat	Pneumonia of left lower lobe	No	No	Azithromycin	NA
19	8.7/F	Thrombocytopenia, seizure disorder, pelvic floor dysfunction, developmental delay	Cough, fever	*M. pneumoniae* pneumonia, acute respiratory failure	Yes	Yes	Azithromycin	Improved
20	9.1/M	None	Shortness of breath, cough	*M. pneumoniae* pneumonia, dyspnea	Yes	No	Azithromycin	Presented to an outside hospital 1 week after discharge for shortness of breath
21	9.1/M	None	Cough, fever	Pneumonia	No	No	Azithromycin	NA
22	10.6/M	None	Fever, cough	*M. pneumoniae* infection	No	No	Azithromycin	NA
23	14.8/F	None	Fever, cough, congestion, shortness of breath	Pneumonia of both lower lobes	No	No	Azithromycin followed with levofloxacin	Admitted at day 4 of therapy due to worsening respiratory distress
24	15.7/F	None	Sore throat, cold	URI	No	No	Azithromycin	NA

*ICU, intensive care unit; NA, not available; URI, upper respiratory tract infection.
